# Gp05, a Prophage-Encoded Virulence Factor, Contributes to Persistent Methicillin-Resistant Staphylococcus aureus Endovascular Infection

**DOI:** 10.1128/spectrum.00600-23

**Published:** 2023-06-26

**Authors:** Yi Li, Fengli Zhu, Adhar C. Manna, Liang Chen, Jason Jiang, Jong-In Hong, Richard A. Proctor, Arnold S. Bayer, Ambrose L. Cheung, Yan Q. Xiong

**Affiliations:** a The Lundquist Institute for Biomedical Innovation at Harbor-UCLA Medical Center, Torrance, California, USA; b Department of Microbiology & Immunology, Geisel School of Medicine at Dartmouth, Hanover, New Hampshire, USA; c Center for Discovery and Innovation, Nutley, New Jersey, USA; d Department of Chemistry, Seoul National University, Seoul, South Korea; e Department of Medicine, University of Wisconsin School of Medicine and Public Health, Madison, Wisconsin, USA; f Department of Medical Microbiology and Immunology, University of Wisconsin School of Medicine and Public Health, Madison, Wisconsin, USA; g David Geffen School of Medicine at UCLA, Los Angeles, California, USA; Riverside University Health System, Medical Center—University of California

**Keywords:** MRSA, phage-encoded virulence factor, vancomycin persistence, endovascular infection

## Abstract

Persistent methicillin-resistant Staphylococcus aureus (MRSA) endovascular infections represent a serious public health threat. We recently demonstrated that the presence of a novel prophage ϕSA169 was associated with vancomycin (VAN) treatment failure in experimental MRSA endocarditis. In this study, we assessed the role of a ϕSA169 gene, ϕ80α_*gp05* (*gp05*), in VAN-persistent outcome using *gp05* isogenic MRSA strain sets. Of note, Gp05 significantly influences the intersection of MRSA virulence factors, host immune responses, and antibiotic treatment efficacy, including the following: (i) activity of the significant energy-yielding metabolic pathway (e.g., tricarboxylic acid cycle); (ii) carotenoid pigment production; (iii) (p)ppGpp (guanosine tetra- and pentaphosphate) production, which activates the stringent response and subsequent downstream functional factors (e.g., phenol-soluble modulins and polymorphonuclear neutrophil bactericidal activity); and (iv) persistence to VAN treatment in an experimental infective endocarditis model. These data suggest that Gp05 is a significant virulence factor which contributes to the persistent outcomes in MRSA endovascular infection by multiple pathways.

**IMPORTANCE** Persistent endovascular infections are often caused by MRSA strains that are susceptible to anti-MRSA antibiotics *in vitro* by CLSI breakpoints. Thus, the persistent outcome represents a unique variant of traditional antibiotic resistance mechanisms and a significant therapeutic challenge. Prophage, a critical mobile genetic element carried by most MRSA isolates, provides their bacterial host with metabolic advantages and resistance mechanisms. However, how prophage-encoded virulence factors interact with the host defense system and antibiotics, driving the persistent outcome, is not well known. In the current study, we demonstrated that a novel prophage gene, *gp05*, significantly impacts tricarboxylic acid cycle activity, stringent response, and pigmentation, as well as vancomycin treatment outcome in an experimental endocarditis model using isogenic *gp05* overexpression and chromosomal deletion mutant MRSA strain sets. The findings significantly advance our understanding of the role of Gp05 in persistent MRSA endovascular infection and provide a potential target for development of novel drugs against these life-threatening infections.

## INTRODUCTION

Methicillin-resistant Staphylococcus aureus (MRSA) is a predominant cause of life-threatening endovascular infections (e.g., bacteremia and infective endocarditis [IE]) (1, 2). Treatment of these syndromes using standard-of-care anti-MRSA antibiotics (e.g., vancomycin [VAN] and daptomycin [DAP]) results in unacceptably high treatment failures and mortality rates (~30%), even with infections caused by *in vitro* VAN/DAP-susceptible MRSA strains as defined by Clinical and Laboratory Standards Institute (CLSI) breakpoints (3, 4). These treatment failures represent a vital variant of the traditional antibiotic resistance mechanism. The current studies focus on the unique mechanism(s) of a novel prophage-encoded virulence factor, Gp05, in VAN-persistent outcomes in MRSA endovascular infection.

Prophages constitute one of the primary sources of genetic diversity that provide their bacterial hosts, including S. aureus, with metabolic advantages and resistance mechanisms to survive in harsh environments (5–7). Our recent research showed a critical role of a novel prophage, ϕSA169, in persistent MRSA endovascular infections (8, 9). ϕSA169 was initially discovered in a clinical MRSA isolate (300-169) from a patient with persistent MRSA bacteremia (PB; defined as ≥5 days of positive blood cultures despite appropriate antibiotic therapy [3]); this prophage was not found in a genetic background-matched (clonal complex 45 [CC45], *agr* I, and SCC*mec* IV) resolving MRSA bacteremia (RB) isolate (301-188) (9). RB is defined as initial MRSA bacteremia resolved within 2 to 4 days of antibiotic therapy (3). We demonstrated that similar to the donor PB isolate, the ϕSA169-lysogenized RB strain variant exhibited the same well-defined *in vitro* phenotypic and genotypic signatures related to the persistent outcome and *in vivo* VAN treatment failure in an experimental IE model (8). However, the mechanism of ϕSA169 in mediating VAN persistence represents a critical gap in knowledge.

Gp05 (55 amino acids [aa]) is a ϕSA169-encoded element, and its function(s) is incompletely defined. Genomic analyses using the published genome databases from the National Center for Biotechnology Information (NCBI) to align nucleotide sequences of *gp05* (168 bp) demonstrated that 26.5% (35 of 132) of the S. aureus prophages, including ϕSA169, ϕ80α, ϕ53 and ϕ11, carry *gp05* (identity ≥95%) (10, 11). In addition, 77.9% of S. aureus strains (10,606 of 13,622) and 83.4% of MRSA strains (8,079 of 9,685) strains show ≥95% nucleotide identity with the *gp05* sequence (10). These data suggest that *gp05* is a highly conserved genetic element in S. aureus strains, implying that it may play an essential role in S. aureus pathogenesis and antibiotic treatment outcomes.

In this study, we showed that Gp05 results in the downregulation of the tricarboxylic acid (TCA) cycle activity, a major energy-yielding metabolic pathway, which may create metabolic and/or cellular stresses to stimulate *sigB* (a global stress responder in S. aureus) activity, as well as stringent response (SR), a broadly conserved bacterial stress response that controls adaptation to nutrient deprivation promoting an MRSA adaptive response to survive antibiotic exposure and to evade innate host defenses during infections. We further demonstrated that animals infected with the *gp05* overexpression strain persisted to VAN treatment in an experimental IE model. In contrast, animals infected with a *gp05* deletion mutant of a clinical PB MRSA strain (300-169) were hypersusceptible to VAN treatment compared with untreated control animals in the IE model. These data suggested that Gp05 is a significant virulence factor in mediating persistent MRSA endovascular infection outcomes through its effect on the intersections of MRSA virulence determinants, host defenses, and antibiotic activity.

## RESULTS

### Gp05 has no effect on VAN MICs.

**FIG 1:**
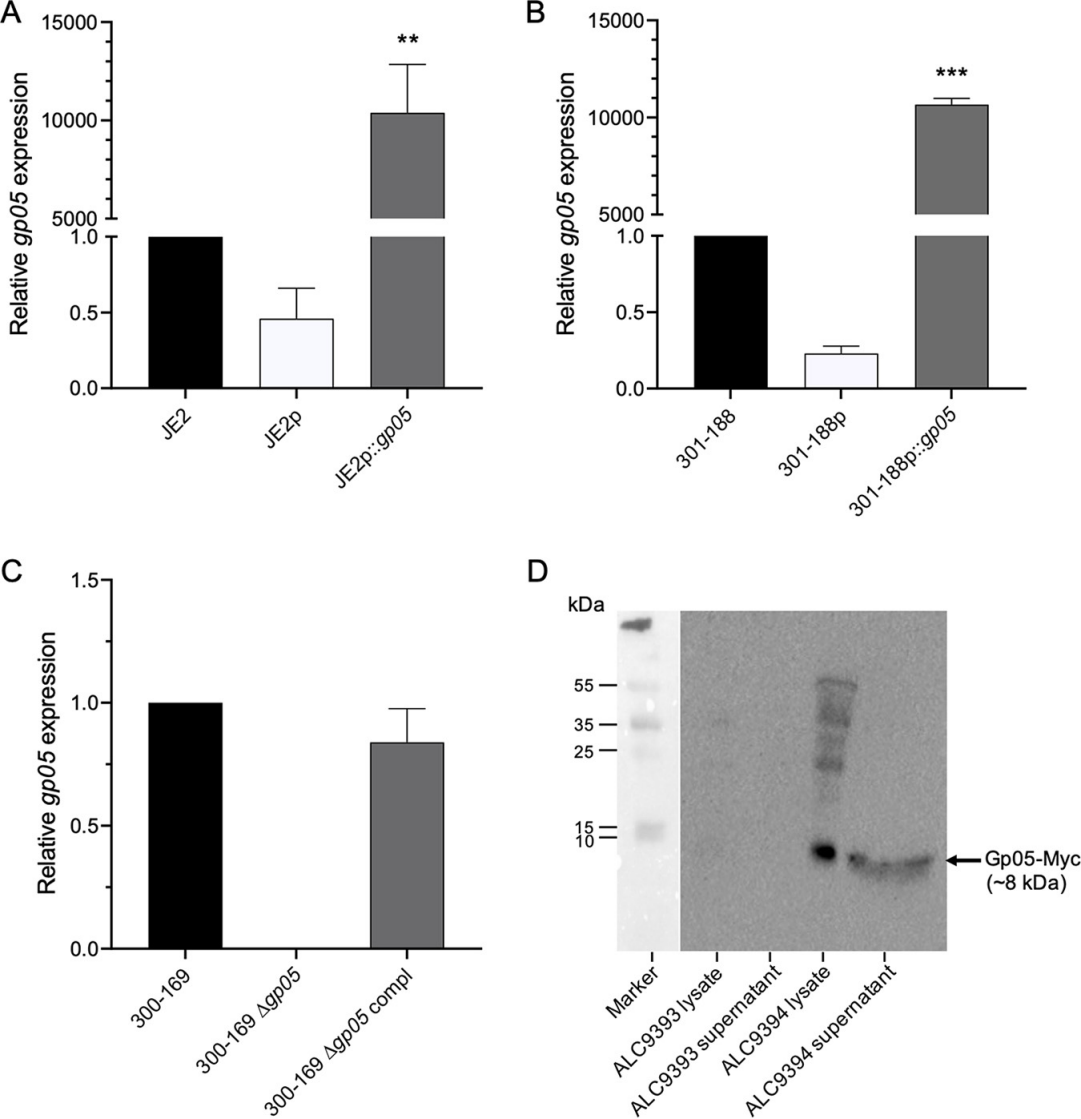
Expression of *gp05* in JE2 (A), RB MRSA 301-188 (B), and PB MRSA 300-169 (C) strain sets. Expression of Gp05 by Western blotting (D). *, *P < *0.05; **, *P < *0.01 versus WT and vector control and *gp05*-complemented strains.

Prior to initiating the studies, we examined the expression of *gp05* in our study strain sets. As expected, the *gp05* overexpression strains (JE2p::*gp05* and 301-188p::*gp05*) exhibited significantly higher *gp05* expression than their isogenic wild-type (WT) and vector control strains did (Fig. 1A and B). In addition, the *gp05* deletion strain had no *gp05* expression, while its WT and *gp05* complemented strains had significantly higher *gp05* expression (Fig. 1C). All the study strains were susceptible to VAN, with MICs of ≤2.0 μg/mL (Table 1) based upon the CLSI breakpoints (4). In addition, the *gp05* overexpression and deletion strains had VAN MICs identical to those of their respective WT and vector control or *gp05*-complemented strains (Table 1).

**TABLE 1:** Strains and plasmids used in this study

Strains or plasmids	Description	VAN MIC (μg/mL)	Reference or source
MRSA strains			
JE2	CA-MRSA, a USA300 LAC derivative cured of its plasmids	1.0	52
JE2p	JE2 with pSK236	1.0	This study
JE2p::*gp05*	JE2 with pSK236::*gp05*	1.0	This study
301-188	RB-MRSA, *agr*-*I* SCC*mec* IV CC45	1.0	4
301-188p	301-188 with pSK236	1.0	This study
301-188p::*gp05*	301-188 with pSK236::*gp05*	1.0	This study
300-169	PB-MRSA, *agr-I* SCC*mec* IV CC45	1.0	4
300-169 Δ*gp05*	300-169 with chromosomal deletion of *gp05*	1.0	This study
300-169 Δ*gp05* complemented	300-169 Δ*gp05* with pCL84::*gp05*	1.0	This study
E. coli strain			
IM08B	General shuttle *dam* methylation strain between E. coli and S. aureus; *dam^+^* Δ*dcm hsdRM*-2CC8 Δ*recA*		54
Plasmids			
pSK236	Shuttle vector containing pUC19 cloned into the HindIII site of pC194; Chlor^r^ Ap^r^		53
pMAD-X	β-Gal, Chlor^r^; modified pMAD with *cat* gene by removing *erm* gene		56
pCL84	Single-copy integration vector for S. aureus; Tc^r^ Sp^r^ *attP* pGB2 *ori*		57

### Gp05 is a secreted protein.

Both supernatant and intracellular lysate of 300-169 harboring pSK236::*gp05-myc* construct (ALC9394) displayed production of Gp05-Myc, while 300-169 with empty vector (ALC9393) had no Gp05 band detected by Western blotting (Fig. 1D). These results suggest that Gp05 is a secreted protein produced in the MRSA strain containing the *gp05* gene.

### Gp05 significantly reduces TCA cycle activity.

**FIG 2:**
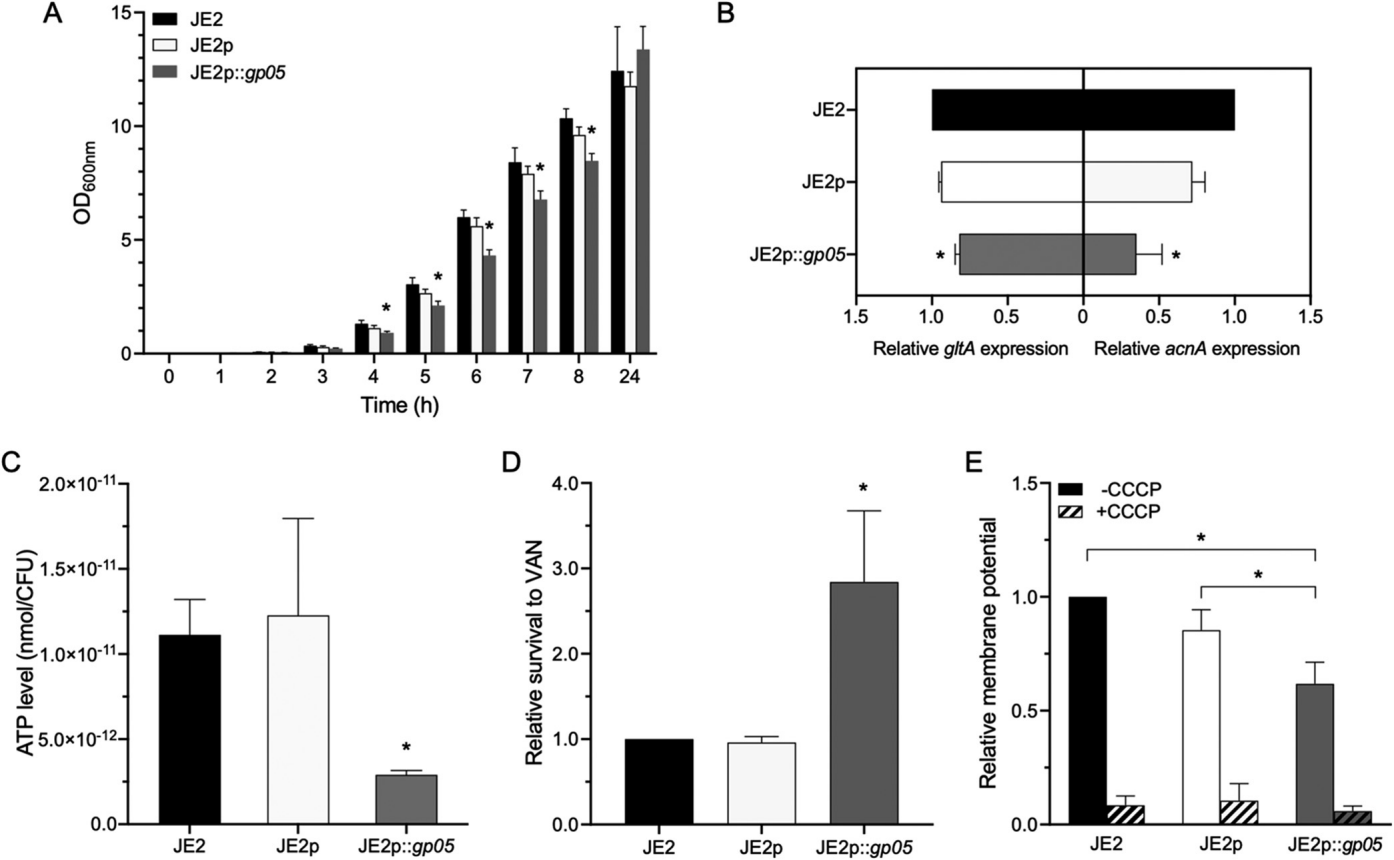
Growth curves (A), expression of key TCA cycle genes *gltA* and *acnA* (B), intracellular ATP levels (C), susceptibility to VAN killing under conditions mimicking scenarios in humans (D), and membrane potential (E) in the JE2 strain set. *, *P < *0.05 versus the WT and vector control strains.

We first tested the growth curve of the study strain set and found that the *gp05* overexpression strain of JE2 grew significantly slower during the 4 to 8 h of incubation time than its WT and vector control strains did (Fig. 2A). This reduced growth rate in the *gp05* overexpression strain might be due to insufficient energy supply (e.g., reduced intracellular ATP level). Since the TCA cycle is a central metabolic pathway that produces ATP for bacteria (12, 13), we tested the expression of two key genes in the TCA cycle (*gltA* and *acnA*) and the intracellular ATP level. A significantly lower expression of *gltA* (also called as *citZ*; this gene encodes a citrate synthase converting acetyl coenzyme A [acetyl-CoA] to citrate) (14) and *acnA* (also called as *citB*; this gene encodes an aconitase converting citrate to isocitrate) (14) was observed in the *gp05* overexpression strain versus its WT and vector control strains (Fig. 2B). Consistent with the reduced TCA cycle gene expression described above, the intracellular ATP level was ~70% lower in the *gp05* overexpression strain than in its isogenic WT and vector control strains (Fig. 2C). Importantly, it has been reported that a reduced intracellular ATP level is associated with antibiotic susceptibility and persistence (15, 16); however, we found that the isogenic *gp05* strain set has identical VAN MICs. Thus, we examined *in vitro* VAN killing activity under conditions mimicking the scenario in human endovascular infection (e.g., target tissue MRSA counts in IE and VAN trough serum levels following standard human dose regimens) (4). We showed that the *gp05* overexpression strain of JE2 exhibited a significantly higher survival rate following VAN exposure than its WT and vector control strains did (Fig. 2D). Besides intracellular ATP levels, membrane potential (Δψ; important for the proton motive force [PMF] across the cell membrane) is also involved in antibiotic susceptibility, and lower Δψ is produced by interrupting the TCA cycle (17). Thus, we tested the impact of *gp05* on Δψ and found a significantly reduced Δψ in the *gp05* overexpression strain versus those in its WT and vector control strains (Fig. 2E). As expected, the addition of PMF inhibitor carbonyl cyanide 3-chlorophenylhydrazone (CCCP), serving as a control, dissipated the Δψ in all study strains (Fig. 2E).

### Reduced TCA cycle activity by Gp05 induces the stress responsive alternative sigma factor (SigB), subsequently increases STX pigment production, and impairs neutrophil killing via antioxidant activity.

**FIG 3:**
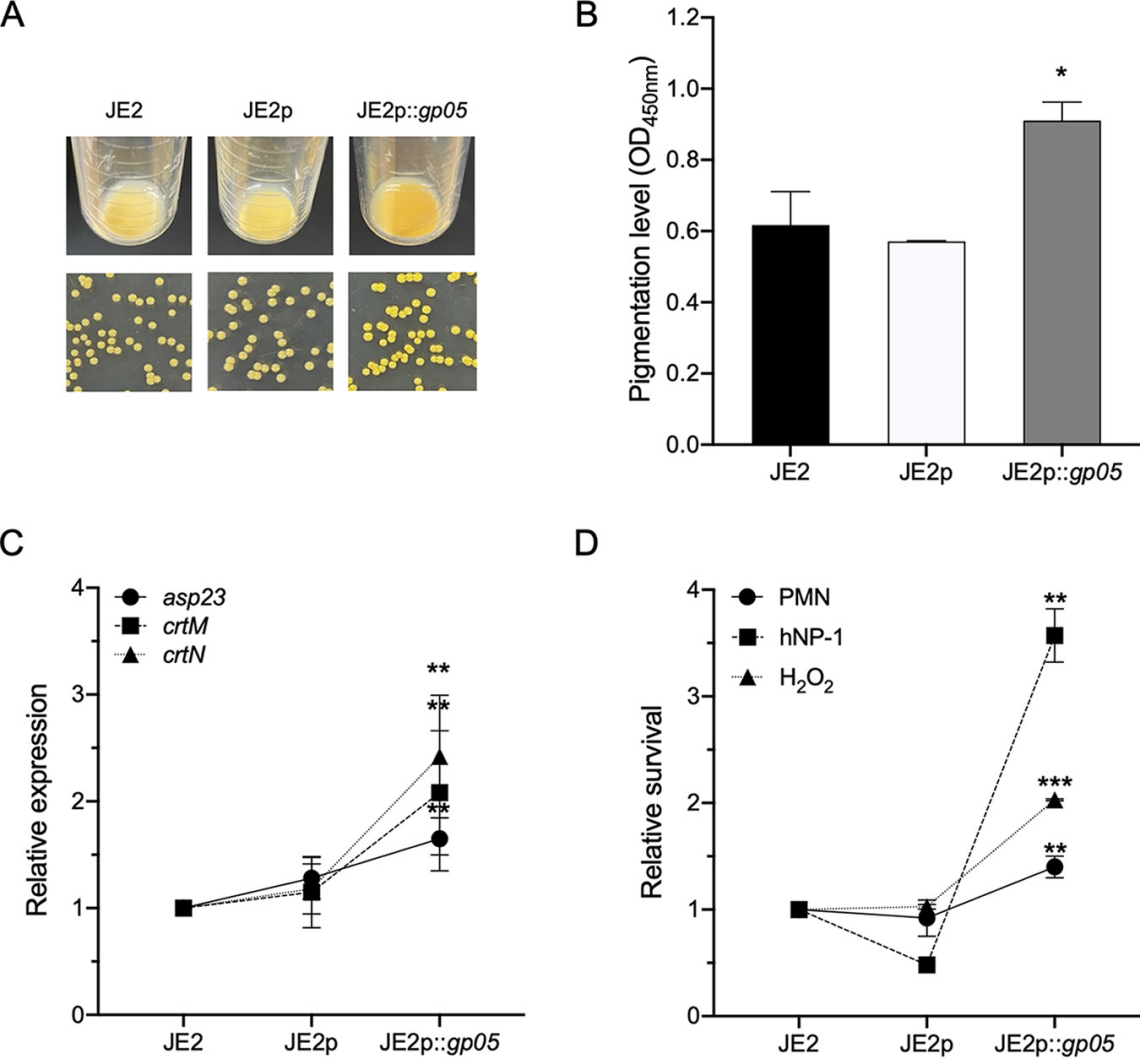
Pigmentation in 24-h culture tubes and on TSA plates (A), pigmentation levels (B), expression of *asp23*, *crtM*, and *crtN* (C), and survival rates after exposure to human PMNs, hNP-1, and H_2_O_2_ (D) in the JE2 strain set. *, *P < *0.05; **, *P < *0.01; ***, *P < *0.001 versus the WT and vector control strains.

We noticed that *gp05* overexpression in strain JE2 yielded an obviously more golden yellow color than its WT and vector control strains (Fig. 3A). The golden yellow color in S. aureus is invariably associated with accumulated staphyloxanthin (STX) (18). Thus, we quantified the production of STX in this study strain set. Significantly higher STX production resulting from *gp05* overexpression in strain JE2 was observed than those in its isogenic WT and vector control strains (Fig. 3B). To explore the mechanism of Gp05-stimulated hyperproduction of STX, we examined the expression of the SigB-activated gene as well as the STX biosynthesis-related genes under SigB control (e.g., *sigB*, *crtM*, and *crtN*). In accordance with the STX production results, the *gp05* overexpression strain showed significantly higher *asp23* (a target for *sigB* activation), *crtM*, and *crtN* expression than those of its WT and vector strains (Fig. 3C). Of note, STX is recognized as an important factor for S. aureus survival in the presence of polymorphonuclear neutrophils (PMNs) related to intracellular killing by reactive oxygen species (ROS) and/or granules containing host defense peptides (19–21). Thus, we studied the effect of Gp05 on the killing activities of PMNs and human neutrophil peptide 1 (hNP-1; a PMN granule-stored cationic antimicrobial peptide) and the ROS (e.g., hydrogen peroxide [H_2_O_2_]). These studies demonstrated that *gp05* overexpression impaired bactericidal activity of human PMNs, hNP-1, and H_2_O_2_ versus its WT and vector control strains (Fig. 3D).

### Lower TCA cycle activity by Gp05 enhances (p)ppGpp, which activates SR and PSMs.

**FIG 4:**
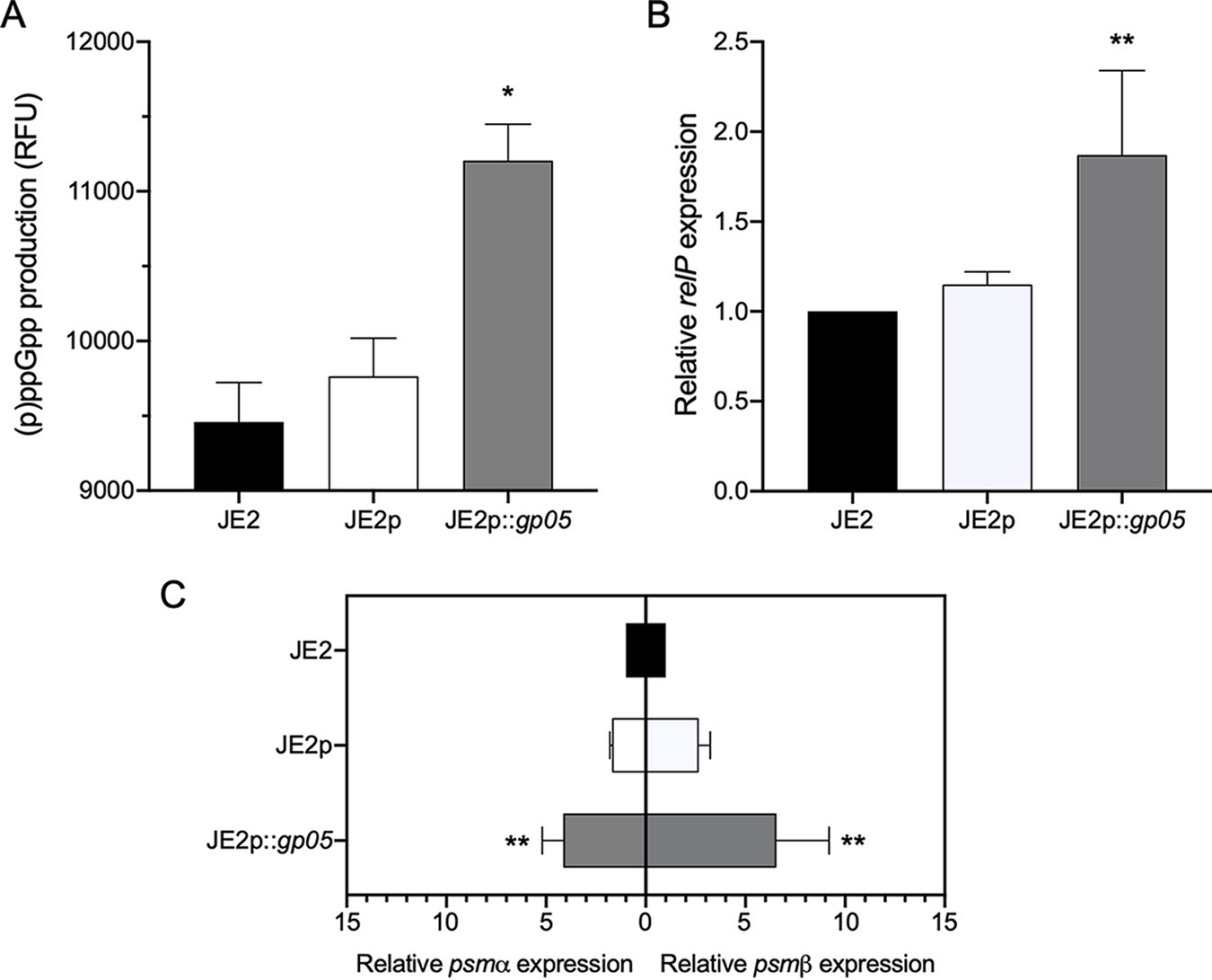
Intracellular (p)ppGpp levels (A), expression of *relP* (B), and expression of *psmα1-4* and *psmβ1*,*2* (C) in the JE2 strain set. *, *P < *0.05; **, *P < *0.01 versus the WT and vector control strains.

Intracellular accumulation of (p)ppGpp triggers the stringent response (SR) and its downstream virulence factors, including phenol-soluble modulins (PSMs) (22, 23). Cytotoxic PSMs promote S. aureus-mediated lysis of PMNs and reduce PMN bactericidal activity (22, 23), which likely contributes to the persistent outcomes. Thus, we investigated the interrelationship of Gp05, (p)ppGpp, the SR, and PSMs in driving PB outcomes. We found that the overexpression of *gp05* yielded significantly increased (p)ppGpp production (Fig. 4A) and expression of *relP*, an important (p)ppGpp synthetase gene (24, 25) (Fig. 4B), and *psmα1-4* and *psmβ1*,*2* transcription (Fig. 4C) versus those in its isogenic WT and vector control strains.

### Gp05 is significantly associated with persistence to VAN treatment in an experimental IE model.

**FIG 5:**
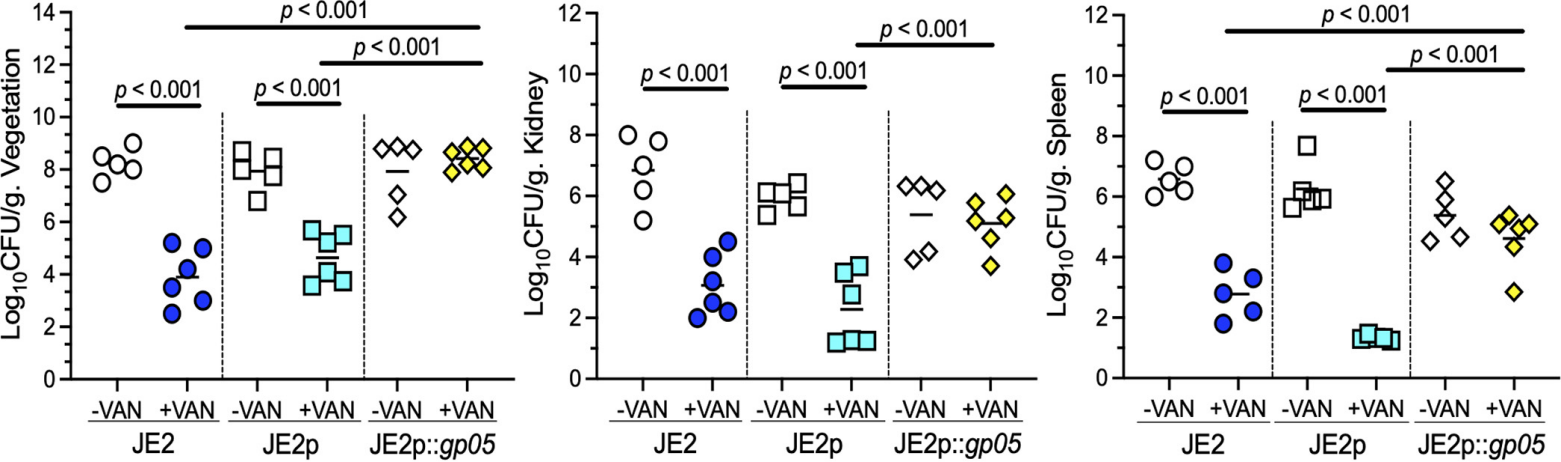
Densities of the JE2 strain set in cardiac vegetation, kidney, and spleen in the IE model with and without VAN treatment. Each symbol represents one animal. Horizontal black bars indicate the mean MRSA densities.

To define the role of Gp05 *in vivo*, we employed a rabbit IE model to study its impact on intrinsic virulence and VAN therapy responsiveness. Notably, without VAN treatment, there were no statistically significant differences in *in vivo* intrinsic virulence among the study strains, based on achievable MRSA counts in cardiac vegetation, kidney, and spleen (Fig. 5). In contrast, MRSA densities in target tissues were significantly higher in VAN-treated animals infected with the *gp05* overexpression strain (JE2p::*gp05*) than in those infected with JE2 WT or vector control strain (Fig. 5). Therefore, overexpression of *gp05* correlated with persistence to VAN treatment in the IE model.

### Overexpression of *gp05* in RB 301-188 has *in vitro* PB-related genotypic and phenotypic profiles similar to those defined in the JE2 strain set.

To further study the impact of overexpression of *gp05* on PB-related *in vitro* profiles, a clinical RB MRSA (301-188 with natural *gp05* negative) strain set was generated. Similar to the findings in the JE2 strain set, overexpression of *gp05* in 301-188 resulted in significantly (i) slower growth during 5 to 7 h of incubation (see Fig. S1A in the supplemental material), (ii) enhanced expression of STX-related genes (*asp23*, *crtM*, and *crtN*) (Fig. S1B), and (iii) increased survival rates in response to PMN, hNP-1, and H_2_O_2_ exposure (Fig. S1C).

### Deletion of *gp05* in the clinical PB MRSA strain 300-169 confirms the role of Gp05 in VAN-persistent MRSA endovascular infection.

**FIG 6:**
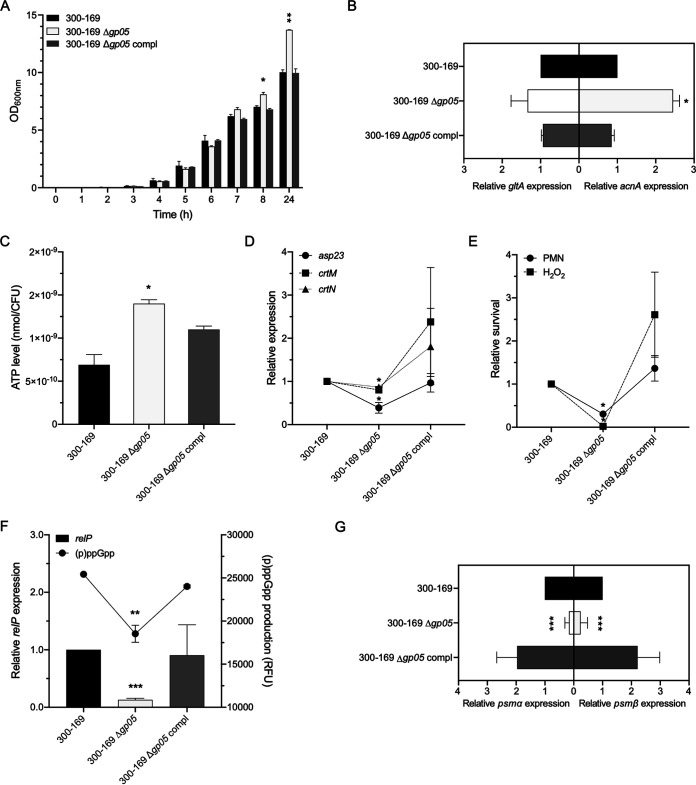
Growth curves (A), expression of *gltA* and *acnA* (B), intracellular ATP levels (C), expression of *asp23*, *crtM*, and *crtN* (D), survival rate after exposure to human PMNs or H_2_O_2_ (E), intracellular (p)ppGpp level and expression of *relP* (F), and expression of *psmα1-4* and *psmβ1*,*2* (G) in the PB MRSA 300-169 strain set. *, *P < *0.05; **, *P < *0.01; ***, *P < *0.001 versus the WT and *gp05*-complemented strains.

To confirm the function of Gp05, we generated an isogenic strain set consisting of a WT clinical PB MRSA strain (300-169, which possesses *gp05* in its genome), its *gp05* chromosomal deletion mutant, and its *gp05*-complemented mutant. We first confirmed that the *gp05* deletion mutant strain had no *gp05* expression, while both the WT and *gp05*-complemented strains expressed *gp05* (Fig. 1C). Importantly, the *gp05* deletion mutant strain verified the impacts of Gp05 seen in the MRSA JE2 background strain set described above on growth rates (Fig. 6A), expression of two critical genes in TCA cycle (Fig. 6B), intracellular ATP levels (Fig. 6C), STX-related gene expression (Fig. 6D), susceptibility to human PMN- and H_2_O_2_-mediated killing (Fig. 6E), intracellular (p)ppGpp levels and *relP* expression (Fig. 6F), and *psmα1-4* and *psmβ1*,*2* expression (Fig. 6G).

**FIG 7:**
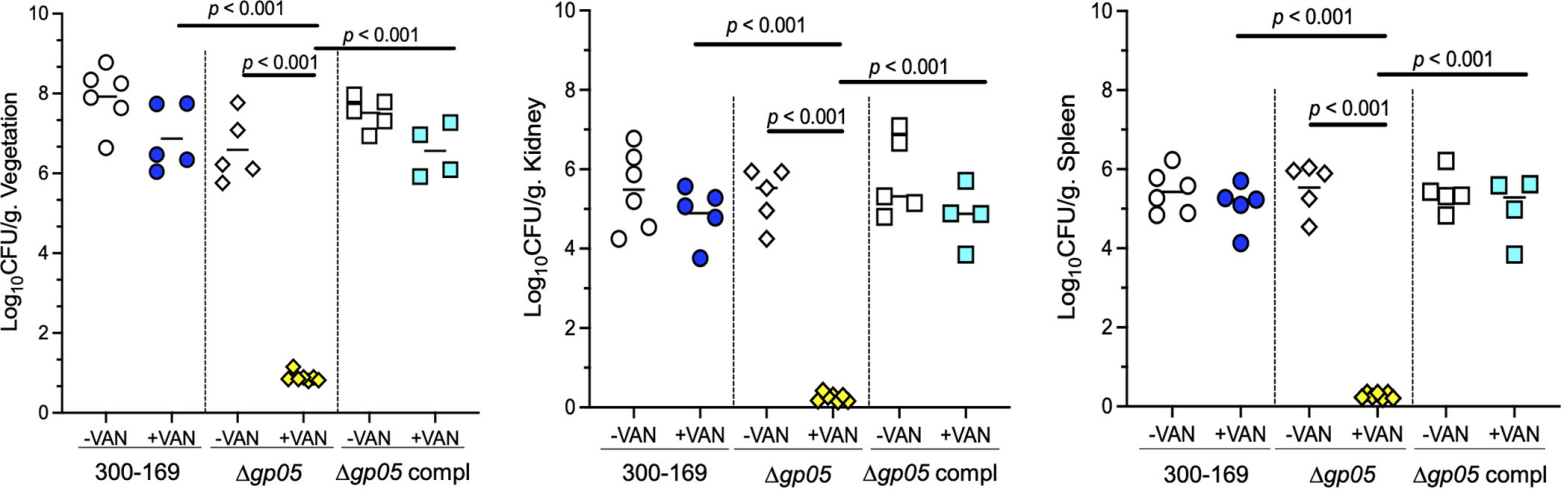
Densities of the 300-169 strain set in cardiac vegetation, kidney, and spleen in the IE model with and without VAN treatment. Each symbol represents one animal. Horizontal black bars indicate the mean MRSA densities.

Of great significance, animals infected with the *gp05* deletion mutant were hypersusceptible to VAN treatment in the experimental IE model, with nearly 100% of target tissues rendered culture negative; in contrast, animals infected with its isogenic WT or *gp05*-complemented strain were nonresponsive to VAN treatment (i.e., a PB outcome [Fig. 7]). These results were accompanied by identical VAN MICs with the strain set (Table 1). Therefore, the *in vivo* results indicated that deletion of *gp05* in 300-169 led to hypersusceptibility to VAN treatment in the IE model.

## DISCUSSION

Extensive genome variation exists in S. aureus strains, with up to 22% dispensable DNA sequences (26). These genetic diversities are largely due to the acquisition of mobile and integrative genetic elements, including phages, pathogenicity islands, plasmids, conjugative transposons, etc. (27). Sequencing efforts show that most S. aureus isolates carry one to four prophages in their genomes, which can occupy up to 20% of the chromosomal content (28–30). These findings suggest that phages have been successfully distributed within S. aureus populations, and phage-encoded virulence factors may contribute to the pathogenesis and treatment outcomes in S. aureus infections (27, 30, 31). The current study was designed to study the role of a novel phage-encoded virulence factor, Gp05, in persistent MRSA endovascular infections. We demonstrated that Gp05 is a significant virulence determinant contributing to MRSA persistence potentially through its regulation in TCA cycle activity, STX production, the SR and their downstream functionalities (e.g., PSMs, PMNs, ROS, etc.). These results may collectively point to adaptive pathway linking to MRSA survival with pivotal interactions with both the human innate immune system and antibiotics exposures.

Several exciting observations emerged in the current investigations. First, we demonstrated that Gp05 significantly impacted TCA cycle activity. For instance, overexpression of *gp05* led to reduced expression of TCA cycle-related genes (e.g., *gltA* and *acnA*) compared with its isogenic WT and vector control strains. Since the TCA cycle is crucial for ATP production and Δψ maintenance in S. aureus (16, 17), the overexpression of *gp05* yielded the expected decreases in intracellular ATP levels and Δψ. Moreover, reduced intracellular ATP level is correlated with a key metabolic trait evoking the persistence of bactericidal antibiotics among Gram-positive (e.g., S. aureus) (15, 16) and Gram-negative (e.g., Escherichia coli and Salmonella enterica) bacteria (32, 33). Conlon et al. reported that reduced intracellular ATP levels led to increased frequency of persister cell formation in S. aureus (16). In addition, a decline in intracellular ATP levels could decrease the activity of ATP-consuming antibiotic targets, leading to reduced antibiotic killing activity (16, 32). In the current studies, we also tested VAN bactericidal activity under *in vivo*-simulating conditions. Despite identical VAN MICs among the strain set, the *gp05* overexpression strain had significantly higher survival rates during exposure to VAN versus the WT and control strains. Importantly, the deletion of *gp05* in the PB strain verified the key effects described above for Gp05 hyperexpression on TCA cycle activity and ATP levels. As a secreted protein, Gp05 is transported through the cell membrane and might bind to receptors of recipient cells. The potential transmembrane activities of Gp05 might have interactions with transporter systems, including nutrient and iron uptake. Glucose uptake and catabolism, which supply the primary carbon sources (34), are found to affect TCA cycle through the carbon flow in S. aureus (35, 36). Thus, Gp05 might influence the TCA cycle activity by its potential effect on glucose uptake. Nevertheless, future studies are needed to investigate the corresponding mechanisms. Therefore, these results demonstrated a significant intersection among Gp05, key metabolic pathways (TCA cycle activity and ATP generation), and antibiotic susceptibility (VAN) under *in vivo*-mimicking conditions. The net result of this intersection appears to be a “PB outcome.”

In our studies, we demonstrated a significantly higher STX production in the *gp05* overexpression strain than in its isogenic WT and vector control strains. Fernández et al. also illustrated that Gp05 was positively correlated with STX production by lysogenization of *gp05*-containing prophages (e.g., ϕ80α, ϕ11, or ϕ53) in methicillin-susceptible S. aureus (MSSA) strain RN450 (37). Notably, the *gp05* overexpression strains verified the positive impact of Gp05 on STX production. STX is a well-recognized virulence factor that protects S. aureus from host immune defenses (e.g., PMN killing) through its antioxidant property (19, 38). Consistent with this well-defined mechanism, we showed that Gp05 has a positive relationship with STX production and survival of PMN and H_2_O_2_ exposures. The biosynthetic pathway for STX in S. aureus includes a series of genes, including *crtM* and *crtN*, encoding dehydrosqualene synthase and dehydrosqualene desaturase, respectively (19, 39), under the control of the alternative sigma factor SigB (encoded by *sigB*) (40). As expected, in the current studies, a positive correlation was observed between STX production and the expression of STX biosynthesis-related genes (e.g., *asp23* [a surrogate reporter for *sigB* promoter activity], *crtM*, and *crtN*). It is well accepted that S. aureus utilizes SigB, a global stress responder, to cope with a number of distinct environmental stresses to promote persistence (41, 42). In addition, deletion of *crtM* renders S. aureus colorless and more susceptible to killing by PMNs or whole blood (19, 20). Moreover, loss of pigmentation results in a significant decrease in virulence in murine skin abscess or systemic infection models due to S. aureus (19, 21). Thus, the reduced TCA cycle activity and ATP levels mediated by Gp05 may generate environmental stresses to induce SigB activation, subsequently increasing STX biosynthesis and ultimately resulting in MRSA persistence.

In addition, we demonstrated a positive correlation between Gp05 and intracellular levels of the alarmone (p)ppGpp. For instance, the *gp05* overexpression strain had significantly higher (p)ppGpp levels than both the WT and vector control strains did. In contrast, the *gp05* deletion strain had significantly lower (p)ppGpp production than its WT and gp05-complemented strains did. In S. aureus, accumulation of (p)ppGpp triggers the SR system, a highly conserved adaptation mechanism to overcome environmental stresses, which contributes to persistent outcomes (24, 43). In addition, the SR involves virulence, phagosomal escape, and antibiotic resistance in different pathogenic bacteria using highly distinct mechanisms (44–46). In S. aureus, synthesis of (p)ppGpp is accomplished by three enzymes, RelA/SpoT homolog (RSH) enzyme and two small synthetases, RelP and RelQ (24, 25, 47). It has been demonstrated that the bifunctional enzyme RSH is essential for S. aureus survival due to its hydrolase activity (25, 48). Thus, RelP and/or RelQ is active to synthesize (p)ppGpp in S. aureus and RSH is required to hydrolyze these molecules to prevent toxic accumulation of (p)ppGpp (25). We and others previously demonstrated a significant correlation among *relP* expression, intracellular (p)ppGpp levels, and expression of *psmα1-4* and *psmβ1-*2, which might contribute to PMN lysis and *in vivo* VAN persistence in S. aureus (22, 23). Consistent with the previously defined influence of (p)ppGpp on PSM and PMN activity, we found a positive intersection among Gp05, (p)ppGpp production, expression of *relP* and *psm* genes, and survival of PMN exposure using *gp05* overexpression and deletion strain sets. Taken together, the results led us to identify a new link between Gp05 and the SR that is likely crucial to the PB outcomes.

Most importantly, we demonstrated that Gp05 is a significant novel virulence factor contributing to VAN persistence *in vivo* in the experimental IE model. For instance, MRSA densities in the target tissues were significantly higher in VAN-treated animals infected with the *gp05* overexpression strain of JE2 than in those infected with JE2 WT or vector control strains. Of great significance, animals infected with the *gp05* deletion mutant in PB 300-169 strain were hypersusceptible to VAN treatment, while animals infected with its isogenic WT or *gp05*-complemented variant persisted in response to VAN treatment. These results were observed despite identical VAN MICs with the strain sets. It is recognized that no single virulence determinant alone is sufficient to enable the persistence of MRSA. Thus, the effect of Gp05 on the *in vivo* VAN persistent outcome may be due to its combinatorial impact on S. aureus virulence factor mosaics (e.g., TCA cycle activity, STX pigment, and SR) and the host innate immune system (e.g., PMNs).

In the current studies, we also demonstrated that Gp05 is a secreted virulence factor. Protein structure alignment by HHPred predicts that Gp05 is similar to VraX family proteins (49). Yan et al. showed that VraX specifically inhibits the activation of C1 complex and contributes to the pathogenesis of S. aureus (50). In addition, it has been recently reported *vraX* is significantly upregulated when S. aureus is exposed to several antimicrobial agents (e.g., VAN, DAP, and antimicrobial peptides) (51). However, the detailed function of VraX remains unclear.

**FIG 8:**
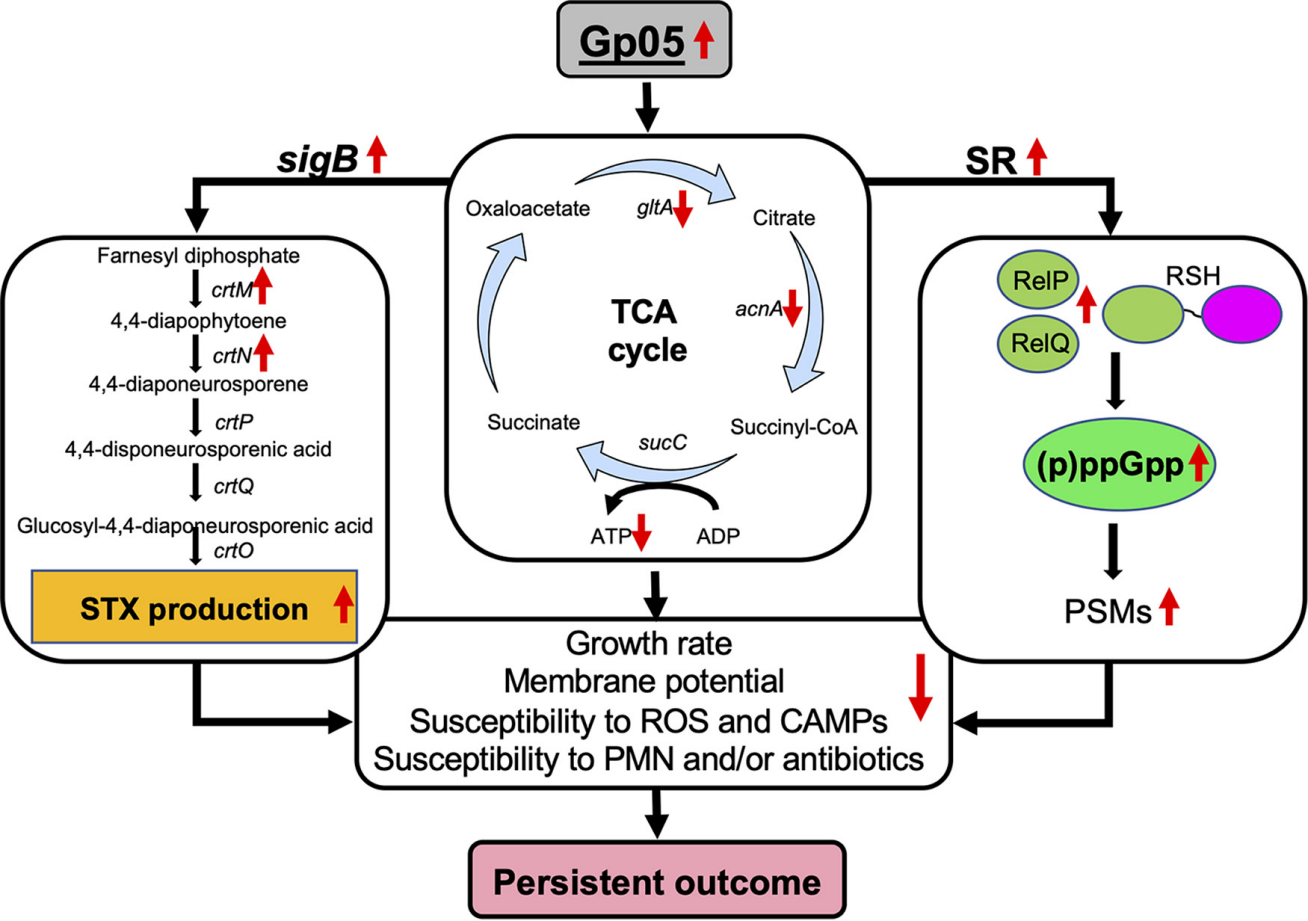
Hypothesized working model of Gp05 in MRSA persistent outcome. Gp05 downregulates TCA cycle activity and decreases ATP production. Reduced TCA cycle activity and ATP levels by Gp05 create metabolic/energy stresses to trigger the alternative sigma factor B (SigB) and stringent response (SR) systems, which are crucial for S. aureus adaptation and persistence through their regulatory effects on downstream virulence factors. For instance, an upregulated SigB system elevates STX production through the *crtMNOPQ* operon, which impairs PMN killing activity via its antioxidant activity. An activated stringent response results in increased production of cytotoxic PSMs to lyse PMNs. In addition, reduced activity of the TCA cycle produces reduced membrane potential, which is associated with increased resistance to cationic antimicrobial peptides (CAMPs), and decreased ATP would reduce the activity of ATP-dependent antibiotic activity. These combinational activities ultimately contribute to *in vivo* persistent outcome.

We propose a hypothesized working model based on the findings in the current study (Fig. 8). In brief, Gp05 impairs TCA cycle activity, which leads to lower ATP production, slower growth, reduced membrane potential, and impaired VAN killing activity. Reduced TCA cycle activity by Gp05 generates metabolic and/or cellular stresses, which, in turn, activate the SigB and SR systems. The upregulated SigB system elevates the production of STX, which reduces host immune system activity (e.g., PMNs) through its antioxidant activity. Activated SR by increased (p)ppGpp level enhances the activity of downstream PSMs and improves microbial survival in the face of the host immune system. Collectively, these combinational phenotypic and genotypic activities caused by Gp05 contribute to the net VAN persistence in MRSA endovascular infection.

We recognize some limitations in the current study. For instance, we only generated a *gp05* deletion mutant of one single PB strain (300-169). Thus, there is a concern that the role of Gp05 may be 300-169 strain specific. Bioinformatic analyses showed that *gp05* is present in about 83% of MRSA strains. It would be interesting to generate *gp05* deletion mutants of additional clinical PB MRSA strains with the most common genetic backgrounds (e.g., CC5 and CC8) in clinical settings associated with complicated endovascular infection to address the strain specificity issue. In addition, we tested only VAN-related persistence. Further studies will test if the role of Gp05 in MRSA persistence is VAN specific by including other standard-of-care anti-MRSA antibiotics (e.g., DAP and ceftaroline).

In summary, our findings unveil that Gp05 is a novel prophage-encoded virulence factor significantly contributing to the PB outcomes in MRSA endovascular infections. Although the mechanisms of Gp05-mediated PB outcomes are not entirely understood, these results significantly advance our understanding of its role in MRSA persistence. Future studies focusing on Gp05 could provide potential targets for novel drug development against these life-threatening infections.

## MATERIALS AND METHODS

### MRSA strains, plasmids, and growth medium.

MRSA strains and plasmids used in this study are listed in Table 1. Overexpression of *gp05* was studied in JE2 (community-associated MRSA [CA-MRSA] USA300 LAC cured of plasmids) (52) and a clinical RB MRSA strain (301-188) (4). In addition, to further study the function of Gp05, a chromosomal deletion of *gp05* in a clinical PB MRSA (300-169) strain was employed. Before all experiments, the *gp05* overexpression mutant and vector control strains of JE2 and 301-188 were recovered and selected on tryptic soy agar (TSA) plates containing 10 μg/mL of chloramphenicol. All study strains were routinely grown at 37°C in tryptic soy broth (TSB; Becton, Dickinson and Company, Franklin Lakes, NJ, USA) or on TSA plates if not otherwise specified.

### Generation of *gp05* overexpression and deletion mutant strains.

To study the function of Gp05, we generated the *gp05* overexpression strain sets by introducing *gp05* under the control of the *sarA* P1 promoter into the JE2 and RB 301-188 strains. In addition, we also constructed a *gp05* deletion in PB 300-169. *gp05* is present chromosomally in JE2 (30) and PB 300-169 (9) but not in RB 301-188 (9). For generating *gp05* overexpression strains, we cloned the *gp05* gene with its ribosome binding sequences under the control of the *sarA* P1 promoter (P1*_sarA_*-*gp05*) into shuttle plasmid pSK236 (53) and selected for ampicillin-resistant colonies in Escherichia coli IM08B (54). The recombinant pSK236 construct, verified by restriction digest and DNA sequencing, was mobilized into the JE2 and 301-188 strains by electroporation. For the deletion of *gp05* in 300-169, we constructed chromosomal deletion of *gp05* in 300-169 using routine procedures as described previously (55). Briefly, a DNA fragment containing 1 kb upstream and downstream of *gp05* was amplified by PCR using chromosomal DNA of 300-169 (Erm^r^) as a template. The DNA fragment was cloned into temperature-sensitive shuttle vector pMAD-X (β-galactosidase [β-Gal], Chlor^r^) (56) and then selected in E. coli IM08B (54) for the correct construct. This cloning construct led to the deletion of the 170-bp *gp05* in an ~2-kb fragment in pMAD-X. After confirmation by DNA sequencing, the recombinant pMAD-X was introduced into the 300-169 strain by electroporation and selected on chloramphenicol (10 μg/mL) and 5-bromo-4-chloro-3-indolyl-β-d-galactopyranoside (X-Gal)-containing plates for blue colonies at 30°C. Plasmid DNA was isolated and authenticated for the presence of DNA fragments in the construct in 300-169. The construction of chromosomal deletion of *gp05* in the 300-169 strain by two-point crossover was performed by a routine procedure as described previously (55). Finally, the mutant clones were verified by chromosomal PCR and DNA sequencing of the PCR product for the deletion of *gp05*. In addition, we complemented the *gp05* deletion mutant strain as a confirmation, using the chromosomal integration vector pCL84 containing *gp05* (57). The expression level of *gp05* in all study strain sets was determined by quantitative real-time PCR (qRT-PCR) using total RNA extracted from overnight cell culture to confirm successful strain constructions (Fig. 1A to C).

### Detection of Gp05 production.

To determine whether Gp05 is a secreted protein, the *gp05* gene along with the promoter region and in-frame *myc* tag at the C-terminal end was amplified, cloned into the pSK236 shuttle vector (53), and introduced into the 300-169 strain. The expression of Gp05-Myc was determined by analyzing both intracellular and extracellular fractions of 300-169 harboring empty pSK236 (ALC9353) or pSK236::*gp05-myc* (ALC9354) grown overnight at 37°C with a mouse monoclonal anti-Myc tag antibody (Cell Signaling Technology, Danvers, MA, USA) by Western blotting (58). Overnight cultures of study strains were prepared. The supernatant and cells were separated and collected by centrifugation. The cell pellets were resuspended in lysis buffer (25 mM Tris-Cl [pH 7.5], 200 mM NaCl, 1 mM EDTA [pH 8.0]) with 0.1-mm glass/zirconia beads and then disrupted in a FastPrep bead-beater (MP Biomedicals, Irvine, CA, USA). The intracellular cell lysates in the supernatant were collected by centrifugation. Proteins from intracellular lysates and filtered culture supernatant were then analyzed using Western blotting as described previously (59) with a mouse monoclonal antibody raised against the Myc tag peptide (Cell Signaling Technology, Danvers, MA, USA). The blot was exposed to ECL substrate (Thermo Fisher Scientific, Waltham, MA, USA) and imaged in a ChemiDoc MP imaging system (Bio-Rad, Hercules, CA, USA).

### RNA isolation and quantification of transcript levels by qRT-PCR.

Total RNA was isolated from study MRSA strains using the method as described previously (60, 61). Briefly, pelleted MRSA cells (~10^9^ CFU) were resuspended in RLT buffer from RNeasy kit (Qiagen, Germantown, MD, USA), then transferred to lysing matrix B tubes (MP Biomedicals, Irvine, CA, USA), and disrupted using FastPrep (Thermo Fisher, Waltham, WA, USA). After centrifugation at 13,000 rpm at 4°C for 10 min, the supernatant was used for RNA isolation according to the manufacturer’s instructions for the RNeasy kit and then treated with TURBO DNase kit (Thermo Fisher) to remove remaining DNA. DNase-treated RNA (1 μg) was transcribed into cDNA using the SuperScript III first-strand synthesis kit (Invitrogen, Waltham, MA, USA) according to the manufacturer’s protocols. qRT-PCR was performed using an ABI Prism 7000 instrument (Applied Biosystems, Waltham, MA, USA) and a SYBR green PCR master kit (Applied Biosystems) (8). Primers used in this study are listed in Table S1. A housekeeping gene, *gyrB*, was used to normalize the transcript quantification. Relative quantification of interesting gene expression was calculated by the threshold cycle (ΔΔ*C_T_*) method and then normalized versus the relative expression level in the WT strain (4).

### Determination of VAN MICs.

VAN MICs of the study strain sets were determined by a standard Etest method according to the manufacturer’s recommended protocols (bioMérieux, La Balme-les-Grottes, France) (62).

### Growth curve.

Overnight cultures of the study strains were washed and adjusted to an optical density at 600 nm (OD_600_) of 1.0 in phosphate-buffered saline (PBS) and diluted 1:100 into 50 mL of fresh TSB in 500-mL Erlenmeyer flasks. The samples were incubated at 37°C with shaking at 200 rpm for 24 h. Cell growth was monitored spectrophotometrically by measuring OD_600_ hourly from 0 to 8 h and then at 24 h (63).

### Quantification of intracellular ATP levels.

Intracellular ATP levels in overnight cultures were quantified by using the Promega BacTiter-Glo kit (Promega, Madison, WI, USA) (60, 64). ATP levels were determined by measuring luminescence levels and comparing them to ATP standards, which are presented as concentrations normalized to the number of CFU.

### *In vitro* VAN killing activity under conditions mimicking those in humans.

Overnight cultured of the study strains were washed, adjusted to an OD_600_ of 1.0 in PBS, diluted 1:10 into cation-adjusted Mueller-Hinton broth (MHB) to achieve an initial inoculum of ~10^8^ CFU/mL (similar MRSA density in cardiac vegetations in the IE), and exposed to VAN at 15 μg/mL (serum trough levels in human with standard VAN treatment in severe MRSA infections) (4) at 37°C, with shaking at 200 rpm overnight. Survival rates were calculated as the ratio of the MRSA surviving cells versus the initial inoculum.

### Quantification of membrane potential.

The membrane potential of the study strains was determined using a BacLight bacterial membrane potential kit (Life Technologies, Carlsbad, CA, USA) (17). In brief, MRSA cultures were adjusted to an OD_600_ of 0.4 in 1 mL of filtered PBS, mixed with 10 μL of 3 mM 3,3′-diethyloxacarbocyanine iodide [DiOC2(3)] fluorescent dye, and incubated at room temperature for 30 min. In parallel, a control with an additional 10 μL of 500 μM proton motive force inhibitor carbonyl cyanide 3-chlorophenylhydrazone (CCCP) was prepared for each sample. The fluorescent signals were determined at an excitation wavelength of 485 nm and an emission wavelength of 528 nm (green fluorescence) and an excitation wavelength of 485 nm and an emission wavelength of 590 nm (red fluorescence) using a BioTek Synergy 2 microplate reader (BioTek Instruments, Winooski, VT, USA). The ratio between red fluorescence and green fluorescence was calculated to indicate membrane potential (17).

### Extraction and quantification of carotenoid pigment production.

Overnight cultures of the study strains were washed, adjusted to an OD_600_ of 1.0 in PBS, and diluted 1:100 with 50 mL of TSB in 500-mL Erlenmeyer flasks. After a 24-h incubation at 37°C with shaking at 200 rpm, MRSA cells were collected and washed with PBS twice. The cell pellets were then resuspended in methanol (3 mL of methanol/1 g of wet cell pellet) and heated in a 55°C water bath for at least 5 min to extract carotenoids (19, 38). Then the extracts were harvested by centrifugation. The OD_450_s of the methanol extracts were determined to quantify the pigment (38).

### PMN bactericidal activity.

Human polymorphonuclear neutrophil (PMN)-mediated killing was conducted based on the method developed in previous studies (22, 65). Exponential-phase (3-h incubation) cultures of the study strains were washed and adjusted to an OD_600_ of 1.0 in Hanks’ balanced salt solution (HBSS; Thermo Fisher Scientific, Waltham, MA, USA). Frozen human neutrophils (Astarte Biologics, Redmond, WA, USA) were thawed in a 37°C water bath and gently resuspended in HBSS. The bacterial suspension was mixed with ~10^6^ CFU/mL of neutrophils to achieve a multiplicity of infection (MOI) ratio of 10:1 (bacteria to PMNs) and incubated at 37°C and 5% carbon dioxide (CO_2_) for 3 h. The bacterial survival was expressed as the percentage of the initial inoculum that survived the PMN exposure.

### *In vitro* susceptibility to hNP-1.

Susceptibility to human neutrophil peptide 1 (hNP-1) was measured by exposing exponential-phase cells (~10^5^ CFU/mL) of the study strains to 6.25 μg/mL of hNP-1 (Vivitide, Gardner, MA, USA) following the method established previously (66, 67). The hNP-1 concentration was selected based on previous pilot studies, in which we identified peptide levels that did not cause rapid killing of MRSA over a 3-h exposure period (data not shown). Survival rates were calculated as the ratio of the number of surviving cells versus the initial inoculum after the 3-h incubation.

### *In vitro* susceptibility to H_2_O_2_.

Susceptibility to reactive oxygen species hydrogen peroxide (H_2_O_2_) was measured by exposing the study strains to H_2_O_2_ (Thermo Fisher Scientific, Waltham, MA, USA) following the method established previously (19). In brief, overnight cultures of the study strains were washed, adjusted to an OD_600_ of 1.0 (~10^9^ CFU/mL), and exposed to H_2_O_2_ (final concentration of 1.5%). After a 1-h incubation at 37°C, residual H_2_O_2_ was quenched by adding catalase (final concentration, 1,000 U/mL). The survival rate was calculated as the ratio of the number of surviving cells versus the initial inoculum.

### Detection of (p)ppGpp levels.

Intracellular (p)ppGpp levels of the study strains were detected by using a fluorescent chemosensor, PyDPA, as previously described (68, 69). In brief, exponential-phase cells (3-h incubation) of the study strains were adjusted to an OD_600_ of 1.0 in PBS and pelleted by centrifugation. This time point was chosen based on our previous study showing that (p)ppGpp affected the early activation of global regulators (23, 60). After resuspending the pellet in 100% methanol to lyse the cells, the supernatant was collected and concentrated using a FreeZone freeze dryer (Labconco, Kansas City, MO, USA). The dried extracts were then resuspended in HEPES buffer (1 mM, pH 7.4, containing 16% [vol/vol] dimethyl sulfoxide) and mixed with PyDPA (40 μM) for 5 min. Fluorescence was measured using an LS-55 fluorescence spectrometer (PerkinElmer, Waltham, WA, USA) with an excitation wavelength of 344 nm and an emission wavelength of 470 nm for (p)ppGpp levels.

### Experimental IE model in rabbits.

To assess the impact of *gp05* on *in vivo* VAN responsiveness, a well-characterized rabbit model of catheter-induced aortic valve IE model was employed (70, 71). At 72 h after aortic catheterization, animals were infected intravenously (i.v.) with the study strains (~10^5^ CFU/animal, a 95% infective dose [ID_95_] previously established) (4, 70). At 24 h after infection, animals were randomly assigned to receive either no therapy (control) or VAN (15 mg/kg of body weight i.v. twice daily for 3 days, a standard effective dose of vancomycin in the experimental IE model caused by vancomycin-susceptible strains) (72, 73). Control animals were sacrificed at 24 h postinfection in order to determine MRSA density in target tissues at the beginning of VAN treatment. VAN-treated animals were euthanized at 24 h after the last treatment to avoid VAN carryover effects. The cardiac vegetations, kidneys, and spleen were removed and quantitatively cultured (4, 60). MRSA counts in the target tissues were calculated as the mean log_10_ CFU per gram of tissue (±standard deviation [SD]). Rabbits were cared for in accordance with the American Association for Accreditation of Laboratory Animal Care criteria. The Institutional Animal Care and Use Committee (IACUC) of the Lundquist Institute at Harbor-UCLA Medical Center approved the animal studies.

### Statistical analysis.

All *in vitro* experiments were performed in triplicates and repeated at least twice. The two-tailed Student *t* test was employed to analyze the *in vitro* data and the *in vivo* MRSA counts in the target tissues (4). *P* values of <0.05 were considered statistically significant.
