# Supplementary material for: Gp05, a Prophage-Encoded Virulence Factor, Contributes to Persistent Methicillin-Resistant Staphylococcus aureus Endovascular Infection
Source: Microbiol Spectr. 2023 Jun 26;11(4):e00600-23. doi: 10.1128/spectrum.00600-23 (PMC10434118; doi:10.1128/spectrum.00600-23)
Supplement: Supplemental file 1 — Supplemental material. Download spectrum.00600-23-s0001.pdf, PDF file, 0.09 MB [file spectrum.00600-23-s0001.pdf]

Supplementary information

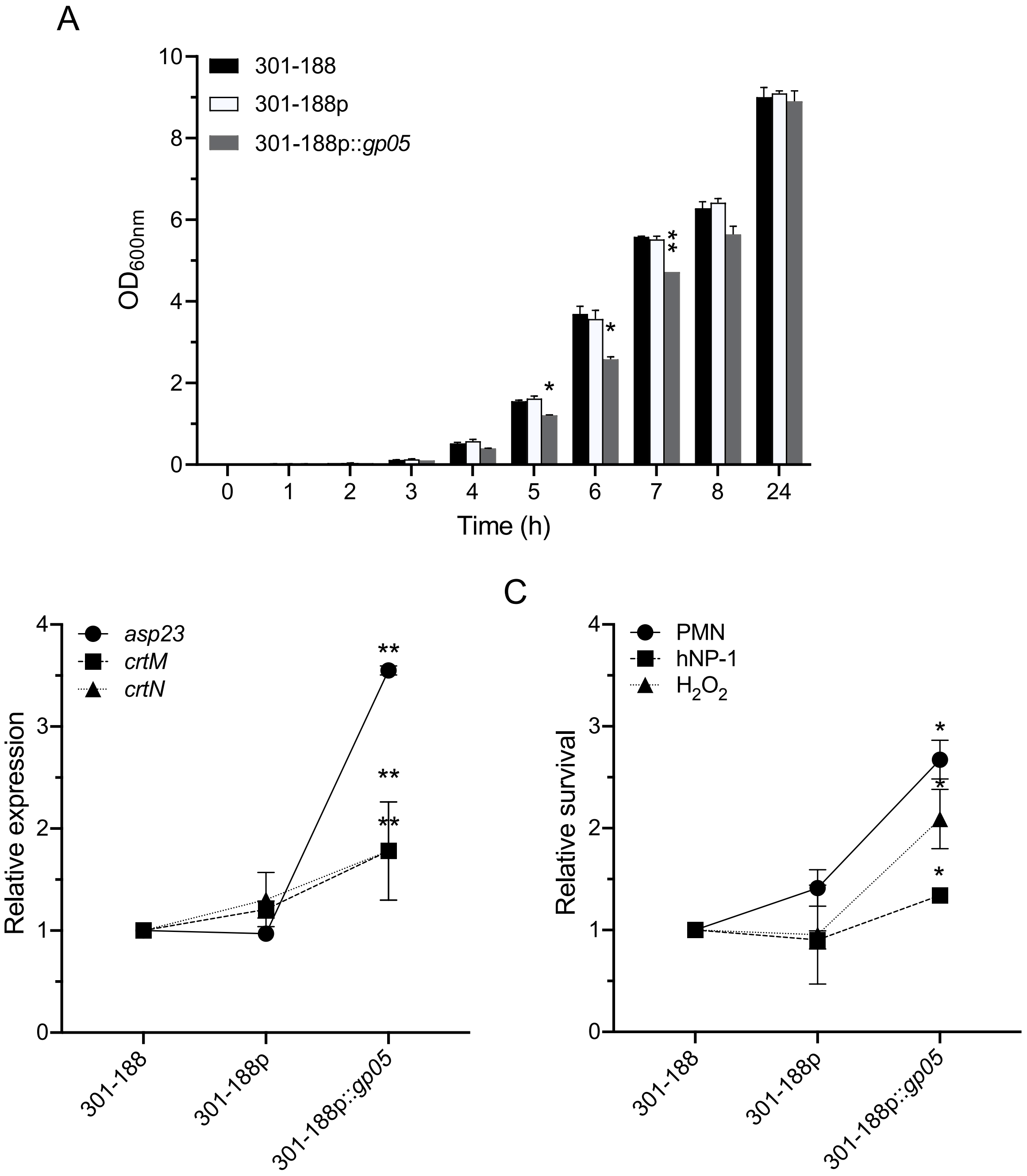

Supplementary Figure 1. Growth curves (A), expression of *asp23*, *crtM* and *crtN* (B), and survival rate with human PMN, hNP-1 or H_2O_2 exposure (C) in RB MRSA 301-188 strain set. * *P* < 0.05, ** *P* < 0.01 vs the WT and vector control strains.

Supplementary Table 1. Primers for RT-qPCR

Name	Sequence (5'-3')	Reference
*asp23*_F	AAAGCAAAACAAGCATACGACAATC	1
*asp23*_R	AGCGATACCAGCAATTTTTTCAAC	
*crtM*_F	TGATGACAGTATAGATGTTTATGG	2
*crtM*_R	ACATGCTGAAGGGCCATCATG	
*crtN*_F	GGTATGTATGGCATGGCTCA	2
*crtN*_R	GGCCCGTTTGAATTTAGGAT	
*relP*_F	CATACTACGCGAATTCCGGC	This study
*relP*_R	TGGAGCGACGTGTGAAAGAA	
psmα*1-4*_F	CATCGTTTTGTCCTCCTG	3
psmα*1-4*_R	TCATCGCTGGCATCATTA	
psmβ*1,2*_F	TAACGCAATTAAAGATACCG	3
psmβ*1,2*_R	TCATGTTGTTGTGCAGCTTG	
*gltA*_F	CCGTAGGTTCTCTGAAAGGGC	4
*gltA*_R	AACATCGTCATAACTTGTTCGTTTG	
*acnA*_F	GGCGCAACAGCAACTGATTT	This study
*acnA*_R	GCACGGTCTGCTAGTGGTAA	
gyrB_F	CGCAGGCGATTTTACCATTA	5
gyrB_R	GCTTTCGCTAGATCAAAGTCG	

References

1. Ster C, Gilbert FB, Cochard T, Poutrel B. Transcriptional profiles of regulatory and virulence factors of *Staphylococcus aureus* of bovine origin: oxygen impact and strain-to-strain variations. *Mol Cell Probes* **19**, 227-235 (2005).

2. Gao P, Davies J, Kao RYT. Dehydrosqualene desaturase as a novel target for anti-virulence therapy against *Staphylococcus aureus*. *mBio* **8**, (2017).

3. Geiger T*, et al.* The stringent response of *Staphylococcus aureus* and its impact on survival after phagocytosis through the induction of intracellular PSMs expression. *PLoS Pathog* **8**, e1003016 (2012).

4. Hartmann T*, et al.* Catabolite control protein E (CcpE) is a LysR-type transcriptional regulator of tricarboxylic acid cycle activity in *Staphylococcus aureus*. *J Biol Chem* **288**, 36116-36128 (2013).

5. Seidl K, Chen L, Bayer AS, Hady WA, Kreiswirth BN, Xiong YQ. Relationship of *agr* expression and function with virulence and vancomycin treatment outcomes in experimental endocarditis due to methicillin-resistant *Staphylococcus aureus*. *Antimicrob Agents Chemother* **55**, 5631-5639 (2011).
